# Local and Global Resting State Activity in the Noradrenergic and Dopaminergic Pathway Modulated by Reboxetine and Amisulpride in Healthy Subjects

**DOI:** 10.1093/ijnp/pyv080

**Published:** 2015-07-25

**Authors:** Coraline D Metzger, Maike Wiegers, Martin Walter, Birgit Abler, Heiko Graf

**Affiliations:** Department of Psychiatry and Psychotherapy, Otto-von-Guericke University, Magdeburg, Germany (Drs Metzger and Walter); Department of Psychiatry, University of Ulm, Germany (Drs Wiegers, Abler, and Graf); Leibniz Institute for Neurobiology, Magdeburg, Germany (Drs Metzger and Walter); Institute of Cognitive Neurology and Dementia Research (IKND), Otto-von-Guericke University, Magdeburg, Germany (Dr Metzger); German Center for Neurodegenerative Diseases (DZNE), Magdeburg, Germany (Dr Metzger).

**Keywords:** Amisulpride, dopamine, noradrenaline, pharmacological resting state fMRI, reboxetine

## Abstract

**Background:**

Various psychiatric populations are currently investigated with resting state fMRI, with the aim of individualizing diagnostics and treatment options and improving treatment outcomes. Many of these studies are conducted in large naturalistic samples, providing rich insights regarding disease-related neural alterations, but with the common psychopharmacological medication limiting interpretations of the results. We therefore investigated the effects of common noradrenergic and anti-dopaminergic medications on local and global resting state activity (rs-activity) in healthy volunteers to further the understanding of the respective effects independent from disease-related alterations.

**Methods:**

Within a randomized, double-blind, placebo-controlled crossover design, we investigated 19 healthy male subjects by resting state fMRI after the intake of reboxetine (4mg/d), amisulpride (200mg/d), and placebo for 7 days each. Treatment-related differences in local and global rs-activity were measured by the fractional amplitude of low frequency fluctuations (fALFF) and resting state functional connectivity (rs-FC).

**Results:**

fALFF revealed alterations of local rs-activity within regions of the core noradrenergic pathway, including the locus coeruleus under reboxetine, correlated with its plasma levels. Moreover, reboxetine led to increased rs-FC between regions within this pathway, i.e. the locus coeruleus, tectum, thalamus, and amygdala. Amisulpride modulated local rs-activity of regions within the dopaminergic pathway, with the altered signal in the putamen correlating with amisulpride plasma levels. Correspondingly, amisulpride increased rs-FC between regions of the dopaminergic pathway comprising the substantia nigra and putamen.

**Conclusion:**

Our data provide evidence of how psychopharmacological agents alter local and global rs-activity within the respective neuroanatomical pathways in healthy subjects, which may help with interpreting data in psychiatric populations.

## Introduction

A variety of investigations conducted with task-related functional magnetic resonance imaging (fMRI) have provided insight into altered brain functioning and divergent neural activations in patients with psychiatric disorders compared to healthy controls. However, this modality is not easily implemented in clinical routines due to its complexity and dependency on the subject`s motivation and performance. Resting state fMRI (rs-fMRI) has been suggested as an alternative task-independent neuroimaging modality suited to discriminate patients with psychiatric disorders from healthy controls (Craddock et al., 2009; Anderson and Cohen, 2013; Nielsen et al., 2013), to predict treatment outcomes (Hadley et al., 2014), and even to distinguish drug-induced side effects (Metzger et al., 2013b). Thus, investigating alterations in baseline brain activity could help investigate the pathophysiological characteristics of psychiatric disorders, with the advantage of an easy application as compared to task-related fMRI (Castellanos et al., 2013). Resting state data are currently acquired in various, mainly naturalistic psychiatric populations within big consortia, aiming to individualize diagnostics, provide treatment options, and improve individual treatment outcomes. These studies provide rich insights into disease-related neural alterations in large samples, but interpretations are limited by the pharmacological treatment that the patients included commonly receive.

The aim of our study was to investigate alterations in spontaneous brain activity by psychotropic medication within a sample of healthy subjects, to allow for separating drug effects from disease-related changes. Previous studies demonstrated alterations in resting state and task-induced activity in healthy males under serotonergic and dopaminergic agents compared to placebos (Abler et al., 2011, 2012; Graf et al., 2013; Metzger et al., 2013b). To further disentangle monoaminergic principles on spontaneous brain activity, we investigated the impact of noradrenergic and anti-dopaminergic medication on local and global spontaneous neural activity.

Local spontaneous low frequency oscillations (LFO) have been related to local neuronal activation (Schölvinck et al., 2010). Such local LFO can be determined by measuring the fractional amplitude of low-frequency fluctuations (fALFF), which describe the relative contribution of local frequency oscillations to the total detected frequency range (Zou et al., 2008). fALFF is therefore primarily considered a marker of local resting state activity. Further, interregional characteristics of rs-fMRI can be described using resting state functional connectivity (rs-FC). Calculated by temporal correlation of LFO between regions, rs-FC provides an indicator of respective synchrony in hemodynamics of two regions (Biswal et al., 1995). rs-FC can therefore be considered a marker of interregional or global resting state activity that indicates interaction between regions. In this study, both local and interregional resting state measures were examined under the noradrenergic antidepressant reboxetine and the atypical antipsychotic drug amisulpride, compared to placebo.

The selective noradrenalin reuptake inhibitor reboxetine (REB) modulates the noradrenergic pathway (NE-pathway; Page, 2003; Hajós et al., 2004) using highly selective blockage of the noradrenaline transporter and consecutive increases of noradrenaline (NE; Invernizzi et al., 2001; Page and Lucki, 2002; Invernizzi and Garattini, 2004; Ortega et al., 2010). Beyond its sympathetic property and peripheral effects within the autonomic nervous system, noradrenaline is one of the classical central neurotransmitters that plays a pivotal role in the pathophysiology of affective disorders (Schildkraut and Kety, 1967; Delgado and Moreno, 2000). There is only one study that previously investigated the impact of REB on rs-FC, revealing reduced striatal-orbitofrontal cortex connectivity compared to placebo (McCabe and Mishor, 2011). However, the intuitive influence of REB on the NE-pathway, as well as local resting state alterations, have not been investigated to date.

The drug amisulpride (AMS) has been shown to act on the dopaminergic pathway (Perrault et al., 1997; Schoemaker et al., 1997; Smeraldi, 1998; Montgomery, 2002; Racagni et al., 2004). It induces pro-dopaminergic effects via modulation of D_2_ autoreceptors at low doses between 50–200mg/d and thereby exerts antidepressant effects, while at high doses it can be used as an antipsychotic (Schoemaker et al., 1997; Montgomery, 2002). The dopaminergic pathway arises from the substantia nigra in the mesencephalon and the ventral tegmental area in the midbrain. It alters basal ganglia functions and terminates in the prefrontal cortex (Nieuwenhuys et al., 2008). AMS has been shown to act on this dopaminergic pathway in animals (Cudennec et al., 1997; Scatton et al., 1997). In humans, AMS shows affinity to these regions, as demonstrated by positron emission tomography (PET) (Vernaleken et al., 2004), and changes task-induced neural activity (Nielsen et al., 2012) and cortical brain perfusion (Viviani et al., 2013). However, alterations in rs-activity under AMS have not yet been described.

This study aimed to investigate local and interregional resting state alterations under both REB and AMS within a randomized, placebo-controlled crossover design. The investigation of drug effects in healthy subjects aims to provide novel aspects to help with interpreting recent rs-fMRI studies on psychiatric disorders, since it allows for disentangling mere drug effects from disease-related alterations.

## Methods

### Participants

A total of 20 healthy male right-handed participants were investigated under 7-day administrations of standard clinical dosages of AMS, REB, and placebo (PLA) in a counterbalanced order separated by washout phases of 2 weeks minimum. Prior to enrolment, subjects underwent a full medical evaluation, including a physical examination, a review of their medical history, and a structured clinical interview for DSM-IV Axis I psychiatric disorders. Each participant received standard blood tests and an electrocardiogram to exclude cardial, renal, or hepatic pathologies. Exclusion criteria encompassed any serious general medical condition, use of any illegal drugs, as well as excessive consumption of caffeine, alcohol (>14 units/week), or nicotine (>15 cigarettes/week). Participants with any kind of current or past history of psychiatric or neurological disorder were also excluded from the study. Upon recruitment, depressive symptoms were assessed using the Center for Epidemiologic Studies Depression-Scale (CES-D; Radloff, 1977) in its German version (Allgemeine Depressions-Skala ADS; Hautzinger and Bailer, 1993). An average CES-D sum score of 8.0 (standard deviation [SD] 6.04) indicated no depressive symptoms prior to the study.

The study was approved by the ethical committee of Ulm University. All volunteers gave written informed consent prior to the study, in accordance with the Declaration of Helsinki. One subject had to be excluded from the study due to glial cerebral pathology (gliotic lesions) revealed by MRI scanning, resulting in a group of 19 participants aged 20–32 (mean age 24.0 years, SD 3.1).

### Study Design and Procedure

**Figure 1.**
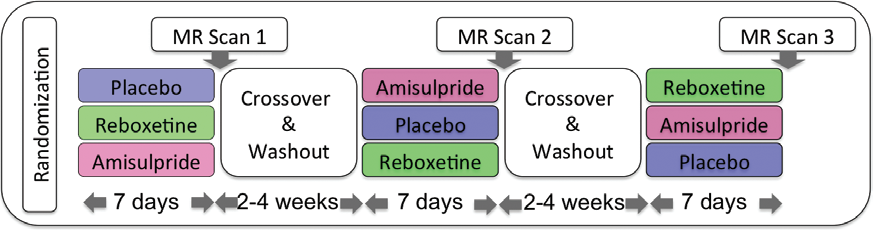
Participants took part in a randomized, placebo-controlled crossover design, taking either reboxetine, amisulpride, or placebo for 7 days with a 14 day washout phase. Resting state scans were acquired after 7 days of drug intake (figure adapted from Abler et al., 2011).

Participants took 200mg amisulpride (100mg twice a day), 4mg reboxetine (2mg twice a day), and placebo (gelatine capsule filled with mannitol, twice a day) for 7 days each in a randomized double-blind crossover design separated by a wash-out phase of at least 2 weeks (Figure 1). fMRI scans took place on the seventh day of drug intake, 2 hours after administration of the last capsule. Each subject was investigated with each of the three substances. Subjects were asked to refrain from caffeine and nicotine on the day of the scan as well as from alcohol throughout the whole time of the study. To confirm drug adherence and exposure, blood samples were collected after each fMRI session (3 hours after last drug intake) and analyzed for drug plasma levels after the study. Drug plasma levels were verified in all subjects, with average values of 137.7ng/ml (SD 54.8) for AMS and of 75.7ng/ml (SD 28.9) for REB.

### fMRI Data Acquisition

Resting state fMRI data were acquired as part of the protocol, including a separate functional task–related MRI experiment and resting state perfusion data, which are reported elsewhere (Viviani et al., 2013; Graf et al., 2015).

Functional and anatomical images were acquired by a 3.0 Tesla Magnetom ALLEGRA Scanner (Siemens). Scanning included acquisition of T1 anatomical volume images (1 x 1 x 1mm voxels), and a 10-minute eyes-closed resting state scan. Task and resting state data were acquired as 23 transversal slices with an image size of 64 x 64 pixels and a field of view of 192mm. A slice thickness of 3mm with a 0.75mm gap resulted in a voxel size of 3 x 3 x 3.75mm. Images were centered on basal structures of the brain, including subcortical regions of interest (basal ganglia, midbrain, and prefrontal regions). T2*-sensitive gradient echo-planar imaging was used to measure changes in Blood-oxygen-level dependent (BOLD) contrast. For the resting state scan, 320 volumes were obtained at a repetition time (TR) of 1500ms (echo time (TE) 35ms, flip angle 90°).

### fMRI Data Analysis

**Figure 2.**
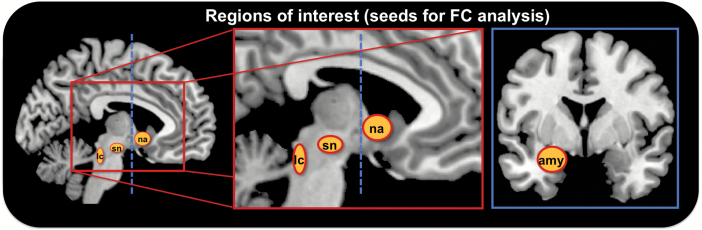
Schematic display of seed regions for resting state functional connectivity analysis. Regions of interest were all left-sided and encompassed the locus coeruleus (lc), the substantia nigra (sn), and the nucleus accumbens (na; saggital view, left and middle), as well as the amygdala (amy; coronal view, right).

Preprocessing of the resting state fMRI data was carried out using the Data Processing Assistant for Resting State fMRI (Chao-Gan and Yu-Feng, 2010; http://www.restfmri.net), which is based on Statistical Parametric Mapping (SPM8, Wellcome Department, http://www.fil.ion.ucl.ac.uk/spm) and the Resting State fMRI Data Analysis Toolkit (Song et al., 2011; http://www.restfmri.net). Preprocessing included slice timing, realignment, and spatial normalization to a standard template (Montreal Neurological Institute) based on Echo Planar Imaging (EPI) templates. Due to partial brain coverage, normalized volumes were masked for those voxels covered in each subject to allow for step performance, especially global mean regression, on equal volumes. Those steps included smoothing with an 8mm full-width at half-maximum Gaussian kernel, removing of linear trends, and filtering with a bandwidth of 0.01–0.08 Hz. The regression of nuisance variables included motion parameters as well as white matter and cerebrospinal fluid signals and global mean regression. Functional connectivity was calculated on the whole brain level for subcortical seed regions within the noradrenergic and dopaminergic pathways. Seed regions were centered on anatomical landmarks based on the T1 template in SPM8. All seed regions were defined as spheres with a radius of 3mm to ascertain spacial specificity to very small anatomical structures. Due to stronger left-lateralized dopaminergic drug effects in previous studies (Abler et al., 2011), regions of interest were defined as left-lateralized spheres and comprised the locus coeruleus (x, -4; y, -30; z, -18) as the core region of the NE-pathway and the substantia nigra (x, -10; y, -14; z, -13) as the core region of the dopaminergic pathway. As we proposed alterations within the noradrenergic and dopaminergic pathways, the amygdala (x, -21; y, 2; z, -21) and the nucleus accumbens (taken from the Harvard-Oxford cortical and subcortical structural atlases, www.cma.mgh.harvard.edu/fsl_atlas.html) were chosen as additional regions that are mainly modulated by both neurotransmitter systems (Nieuwenhuys et al., 2008) and served as two independent regions within these pathways. For the nucleus accumbens, the left-sided region of interest (ROI) consisted of 74 voxels with a voxel size of 2 x 2 x 2mm and was resliced to our original resolution. A schematic display of the seed regions is depicted in Figure 2. All rs-FC maps were z-transformed before further group-level analysis.

The fractional amplitude of low frequency fluctuations (fALFF) was calculated on detrended unsmoothed data of the whole volume, measuring the power within a typical frequency range of 0.01–0.08 Hz divided by the total power in the entire frequency range (Zou et al., 2008). To reduce the global effects of variability across subjects, fALFF of each voxel was divided by the global mean fALFF value within the predefined brain mask, including all voxels covered by each subject. These normalized fALFF values were used for further statistical analysis.

### Second-Level Statistics

A flexible factorial design, as implemented in SPM8, was chosen to test for treatment effects on both local (fALFF) and interregional/global (rs-FC) rs-activity. Analyses of variances (ANOVAs) were applied to assess the main drug effects on fALFF or rs-FC at a conservative significance threshold of *p* < 0.001, adding an additional cluster threshold of more than 10 adjacent voxels for significance (referred to as *p* < 0.001, k = 10), analogous to Metzger et al. (2013b). Post hoc t-tests were performed on the whole brain level to be sensitive to drug-specific effects at the same statistical threshold.

Further, a simple regression analysis was performed to assess the relationship between alterations in fALFF and drug plasma levels. Here, the region with the strongest main effect from the ANOVA f-test was chosen as the ROI, with individual amisulpride and reboxetine blood levels as the regressor.

To be specific to subtle changes in these small regions of interest, correlation analyses were masked with the region of drug-specific main effects at a statistical threshold of *p* < 0.05, uncorrected for the mask. We used MRIcron (http://www.mccauslandcenter.sc.edu/mricro/mricron/index.html) for the creation of displayed figures. Bar plots were created using SPSS Version 15 (IBM Corp.).

## Results

### Local Changes in Resting State Behavior Under REB and AMS

**Figure 3.**
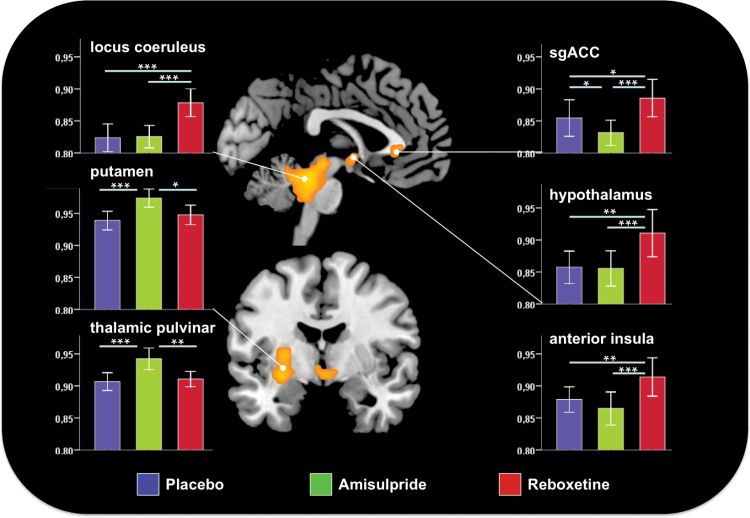
Main effect of drug on the frequency amplitude of low frequency fluctuations (fALFF). Bar diagrams (+/- standard error of the mean) depict mean fALFF under placebo (blue), amisulpride (green), and reboxetine (red). Stars indicate significant changes as revealed by post hoc t-tests (*p < 0.05, **p < 0.01, ***p < 0.001).

The main effects of the drugs on fALFF were found bilaterally in the locus coeruleus, extending to the tectum and ventral cerebellum, the hypothalamus, and the subgenual anterior cingulate cortex (sgACC) as well as in the right ventral anterior insula, left putamen, and right pulvinar (Figure 3, Table S1). Post hoc analysis revealed an increase in fALFF within the putamen and pulvinar under AMS compared to PLA, whereas REB led to an increase of fALFF within the locus coeruleus and the hypothalamus compared to PLA. Comparing the two verum drugs, an increase of fALFF was observed in the sgACC and anterior insula under REB compared to AMS (all *p* < 0.001, k = 10).

### Regression Analyses Between fALFF and Drug Plasma Levels

A positive correlation of REB plasma levels with absolute fALFF under REB was observed within the in locus coeruleus (*p* < 0.031). Moreover, AMS plasma levels were positively correlated with absolute fALFF in the left putamen (*p* = 0.016; Figure S1).

### Global Changes in Resting State Activity Under REB and AMS

#### Main Effects of Drug on rs-FC of the Locus Coeruleus

The ANOVA revealed significant treatment effects on rs-FC between the left locus coeruleus and the bilateral medial and dorsal thalamus, the posterior cingulate cortex (PCC), the cerebellum, and the right amygdala and putamen (*p* < 0.001, k = 10).

Post hoc tests showed mainly increases in rs-FC under AMS compared to PLA on locus coeruleus–seeded connectivity. These increases were found with the putamen, the PCC, and the amygdala (Figure S2, Table S2). Decreased locus coeruleus–seeded connectivity under AMS versus PLA was solely found between the locus coeruleus and the cerebellum.

Locus coeruleus–seeded connectivity under REB as compared to PLA was increased with the medial and dorsal thalamus and the PCC, and decreased with the cerebellum. A detailed summary of results is depicted in Figure S2 and Table S2.

#### Main Effects of Drug on rs-FC of the Amygdala

The main effects of the drugs on the left amygdala-seeded connectivity were observed with the bilateral substantia nigra, tectum, locus coeruleus, thalamus, left hippocampus, left pregenual anterior cingulate cortex (pgACC), and right anterior insula (*p* < 0.001, k = 10). Here, post hoc t-tests revealed an increase in rs-FC between the amygdala and substantia nigra, thalamus, and hippocampus under AMS compared to PLA. Under REB, decreased connectivity of the amygdala with the hippocampus, pgACC, and anterior insula was observed compared to PLA (Figure S2, Table S3).

#### Main Effects of Drug on rs-FC of the Substantia Nigra

An ANOVA revealed treatment effects on substantia nigra–seeded connectivity with the bilateral putamen, hippocampus, and right anterior insula (*p* < 0.001, k = 10). Under AMS, substantia nigra–seeded connectivity was increased with the bilateral putamen, amygdala, and hippocampus compared to PLA. Indeed, REB decreased rs-FC of the substantia nigra with the left putamen compared to PLA (Figure S2, Table S4).

#### Main Effects of Drug on rs-FC of the Nucleus Accumbens

Regarding the left nucleus accumbens seed, the ANOVA analysis revealed treatment effects on rs-FC with the bilateral medial and dorsal thalamus, bilateral sgACC, and left pgACC (*p* < 0.001, k = 10, Figure S2, Table S4).

Under AMS, a decrease of rs-FC was observed between the nucleus accumbens seed with the bilateral sgACC and left pgACC compared to PLA. Under REB, an increase of rs-FC between the left nucleus accumbens and the bilateral medial and dorsal thalamus was found, whereas a decrease in rs-FC of the nucleus accumbens was observed with the bilateral sgACC as well as the left pgACC compared to PLA (Figure S2, Table S5).

## Discussion

We investigated the effects of the noradrenergic antidepressant reboxetine and the atypical antipsychotic drug amisulpride on local (fALFF) and global (rs-FC) rs-activity in healthy male subjects within a randomized, placebo-controlled crossover design. Significant treatment effects were observed for REB, with increased fALFF compared to placebo in core regions within the NE-pathway, e.g. the locus coeruleus and hypothalamus. In contrast, AMS led to an increase in fALFF in the putamen and the thalamic pulvinar, regions associated with the dopaminergic system, compared to PLA. Moreover, alterations in fALFF within the respective neurotransmitter pathways correlated with blood serum levels of REB and AMS.

**Figure 4.**
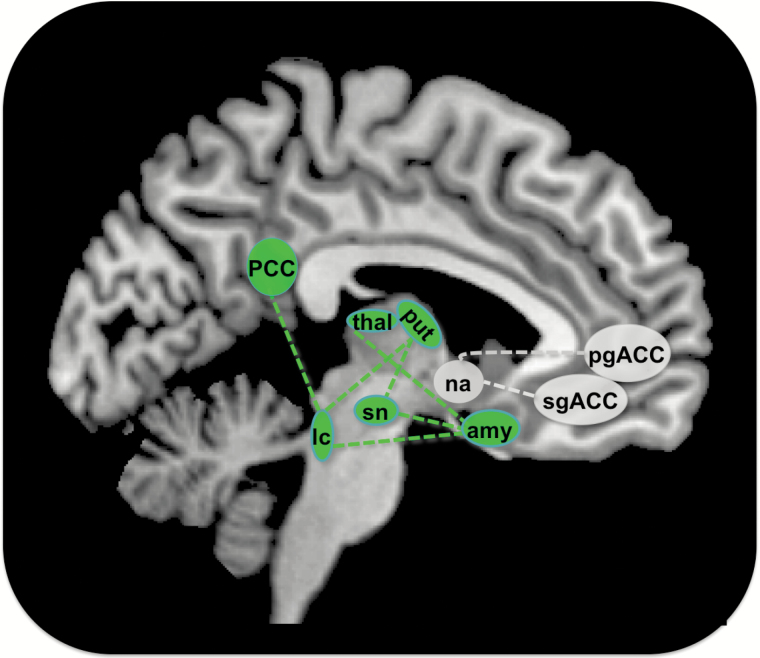
Alterations in resting state functional connectivity (rs-FC) under amisulpride. Amisulpride increased rs-FC between regions depicted as green circles, including the substantia nigra (sn), locus coeruleus (lc), thalamus (thal), putamen (put), and amygdala (amy) on the subcortical and posterior cingulate cortex (PCC) on the cortical level. Green dotted lines indicate an increase in rs-FC between connected regions. Amisulpride decreased rs-FC between the nucleus accumbens (na), the subgenual anterior cingulate cortex (sgACC), and the pregenual anterior cingulate cortex (pgACC), depicted as gray circles. Decreases in rs-FC between regions are indicated by dotted gray lines (*p* < 0.001, k = 10 for all results).

In terms of connectivity, AMS increased rs-FC between regions of the dopaminergic pathway like the substantia nigra, putamen, thalamus and amygdala compared to PLA (Figure 4).

**Figure 5.**
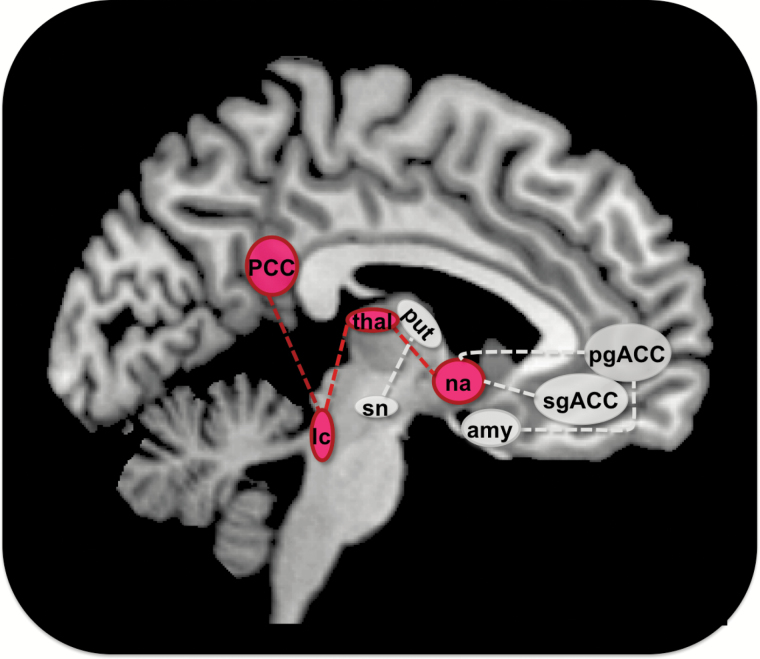
Alterations in resting state functional connectivity (rs-FC) under reboxetine. Reboxetine increased rs-FC between regions, including the locus coeruleus (lc), thalamus (thal), nucleus accumbens (na), and posterior cingulate cortex (PCC), shown in red circles. Red dotted lines indicate an increase in rs-FC between regions. A decrease in rs-FC under reboxetine compared to placebo was observed between the putamen (put), substantia nigra (sn), amygdala (amy), pregenual anterior cingulate cortex (pgACC), and subgenual anterior cingulate cortex (sgACC), displayed as gray circles. Decreases in rs-FC between regions are indicated by gray dotted lines (*p* < 0.001, k = 10 for all results).

In comparison to PLA, REB led to an increase of rs-FC between major regions of the NE-pathway like the locus coeruleus, hypothalamus, and thalamus, and also the nucleus accumbens (Figure 5).

### Modulation of the Noradrenergic Pathway by Reboxetine

As measured by fALFF, local rs-activity increased under REB compared to PLA foremost in the locus coeruleus, tectum, and hypothalamus. Considering the neuroanatomy of the NE-pathway arising from the locus coeruleus to the tectum and hypothalamus via ascending fibers of the dorsal noradrenergic bundle (Nieuwenhuys et al., 2008; Szabadi, 2013), the three observed regions in our study represent “early” regions within the NE system (Szabadi, 2013). Alterations within these regions seem plausible due to the pharmacokinetic properties of REB that increase NE levels by blocking the NE transporter. Notably, increased fALFF within the locus coeruleus was positively correlated with REB blood serum levels. Thus, one might assume that administration of REB in healthy subjects may facilitate neural reactivity within regions of the NE system and therefore increase the variability of fluctuations as measured by fALFF. The same regions showed global resting state alterations under REB compared to PLA, as assessed by rs-FC. An increase in rs-FC was observed between regions of the NE-pathway, including the locus coeruleus, thalamus, hypothalamus, and nucleus accumbens. It is of note that these seed regions for computing rs-FC were chosen independently from the analyses of fALFF and according to anatomical landmarks to avoid circularity (Kriegeskorte et al., 2009, 2010).

An increase in rs-FC between regions indicates higher temporal synchrony of low frequency fluctuations (Biswal et al., 1995). Therefore higher rs-FC between regions of the NE-pathway, as observed under REB, could indicate closer interaction of these regions. One might assume that the increases in extracellular NE as caused by REB (Invernizzi et al., 2001; Invernizzi and Garattini, 2004; Logan et al., 2007; Ortega et al., 2010) account for the increased rs-FC between regions within the NE-pathway. This is supported by animal and human studies that demonstrated increased BOLD activity within these regions under REB, including the hypothalamus and hippocampus, regions that revealed increases in rs-FC between each other under REB in our study (Onur et al., 2009; Brühl et al., 2011; Kukolja et al., 2011; Sekar et al., 2011).

Decreased rs-FC under REB compared to PLA was observed between the amygdala-seed and the pgACC and bilateral insula, as well as for the nucleus accumbens seed with the sgACC. Both sgACC and pgACC are part of the task-negative default-mode network (DMN; Buckner et al., 2008). In depression, rs-FC within the DMN is thought to be increased and has often been linked to strong internal processing, rumination, and attentional bias towards internal action (Greicius et al., 2007). Thus, the decreases in rs-FC under REB between subcortical regions—namely the amygdala and nucleus accumbens—and the DMN in our study may support the hypotheses that antidepressants ameliorate depressive symptomatology by reducing the pre-described “hyperconnectivity” between these regions in depressed patients (Greicius et al., 2007; Berman et al., 2011; Hamilton et al., 2011; McCabe and Mishor, 2011).

### Modulation of the Dopaminergic Pathway by Amisulpride

Compared to PLA, AMS led to an increase in local rs-activity in the putamen. Neural alterations within the putamen by AMS, compared to PLA, are in line with findings on continuous arterial spin–labelling MRI within the same study sample, which revealed a rise of blood perfusion in the putamen under AMS (Viviani et al., 2013). Moreover, alterations within the putamen have also been shown under several anti-dopaminergic medications (Bartlett et al., 1994) and seem in line with the known effect of D2-antagonism that is most pronounced in this region (Holcomb et al., 1996).

Global resting state analyses revealed an increase in rs-FC between the substantia nigra, putamen, nucleus accumbens, thalamus, hippocampus, and amygdala under AMS compared to PLA (Figure S2). Anatomically, these regions have been previously associated with the dopaminergic pathway, arising from the substantia nigra to the ventral tegmental area, nucleus accumbens, and hippocampus and terminating in the prefrontal cortex (Nieuwenhuys et al., 2008). To our knowledge, this is the first study investigating resting state changes under AMS. However, influences of AMS on cerebral metabolism, perfusion, and functional neural activations within the dopaminergic pathway have been reported by invasive studies in animals (Cudennec et al., 1997) as well as in tracer, perfusion, and functional studies in humans (Mehta et al., 2003; La Fougère et al., 2005; Dodds et al., 2009; Viviani et al., 2013). An increase in rs-FC between these regions, as caused by AMS, indicates higher synchrony in low-frequency fluctuations (Biswal et al., 1995). In an earlier study, an increase in perfusion was found in the basal ganglia under AMS, while cortical perfusion was decreased (Viviani et al., 2013). This is in line with our findings of higher fALFF within the basal ganglia under AMS and lower fALFF on the cortical level. As the dopamine receptor is a G-protein coupled receptor, triggering an intracellular signal cascade, this might provide the key to a common concept underlying both observations, but cannot be addressed by our design.

AMS elicits pro-dopaminergic properties (Schoemaker et al., 1997) via modulation of presynaptic D_2_ autoreceptors at low doses between 50–200mg/d, as used in our study. Therefore, an increase in rs-FC between dopaminergic regions under AMS might be interpreted as a closer interaction between regions, positively influenced by a pro-dopaminergic effect of AMS, similarly to increases in fALFF and rs-FC between regions in the NE-pathway under REB. This is supported by a recent study that investigated brainstem dopaminergic pathways using rs-fMRI by administration of the dopamine agonist bromocriptine compared to PLA in healthy subjects (Vytlacil et al., 2014). An increase in mesostriatal rs-FC under the dopaminergic drug was reported within an inverted u-shape relationship. Moreover, decreased rs-FC was found under AMS between nucleus accumbens and sgACC similar as for REB. This effect may contribute to the known antidepressive effect of AMS (Smeraldi, 1998; Montgomery, 2002; Racagni et al., 2004) and might be explained analogous to REB by counteracting a pathological hyperconnectivity in depression (Greicius et al., 2007).

### Drug Effects on Brain Resting State Activity: Possible Consequences for Big Data in Psychiatry

Neural alterations within noradrenergic and dopaminergic pathways have been widely described in psychiatric populations, notably including affective disorders (Delgado and Moreno, 2000; Dunlop and Nemeroff, 2007; Chandley and Ordway, 2012; Price and Drevets, 2012; Savitz and Drevets, 2013), schizophrenia (Kuepper et al., 2012; Whitfield-Gabrieli and Ford, 2012; Lau et al., 2013; Fitzgerald, 2014), attention deficit hyperactivity disorder (reviewed by Posner et al., 2014), and dementia (Sheline and Raichle, 2013; Jacobs et al., 2013). However, a large part of these studies were conducted with patients under psychopharmacological treatment (Cullen et al., 2009; Horn et al., 2010; Lord et al., 2012; van Tol et al., 2014) and the interpretation of their results is therefore limited by the effects of psychotropic medication.

While resting state measurements are independent of task, performance, or compliance, they seem to offer a valuable tool for the investigation of psychiatric populations, where performance or task adherence is sometimes problematic and can therefore result in group effects themselves. Due to these advantages of resting state measurements, more and more large databases of patients’ resting state data are being acquired, promising deeper insights into pathophysiology and treatment decisions, and an increased ability to create accurate predictions (Castellanos et al., 2013). By investigating healthy subjects under psychopharmacological treatment, as in our study, the presented results may facilitate interpretability of rs-fMRI data regarding drug effects in patients rather than disease-related alterations. We provide evidence that REB and AMS alter local and global resting state behavior in regions of the dopaminergic and NE pathways and between these regions and large-scale brain networks like the task-negative default mode network, affected in various psychiatric disorders. Such drug-specific effects might not allow direct interference to drug action in psychiatric populations, but are needed for interpretation when acquiring and analyzing big data in psychiatry (Castellanos et al., 2013).

### Limitations

This study was conducted within a sample of healthy male volunteers to avoid hormonal variations related to the menstrual cycle and their impact on neural activity (e.g. Abler et al., 2013). Thus, our results are limited in their transfer to female samples. While this design was specifically chosen to investigate drug effects on rs-activity independent of disease, divergent drug effects, as well as interactions of drug and disease may be observed in patients and therefore have to be addressed more specifically in future studies.

Moreover, the selection of regions of interest as spheres has to be taken with caution. We chose a very small radius for the selected spheres (3mm) instead of having larger anatomically-shaped ROIs, e.g. for the substatia nigra and the locus coeruleus, in an attempt to be as precise as possible, especially in the small subcortical structures.

By selecting the upper part of the locus coeruleus, we wanted to make sure to catch those parts which can best be normalized, considering deeper structures of the brainstem to be less precise when choosing a standard normalization, as offered by our software, and no specific brainstem normalization, as used by Keren and colleagues (2009).

Further, we are aware that the locus coeruleus and intralaminar parts of the thalamus, as part of the ascending arousal system, are influenced by noradrenaline coding for inner arousal and salience (Nieuwenhuys et al., 2008; Metzger et al., 2010, 2013a). In addition to the amygdala, this system has been recently shown to be affected in its rs-FC by individual heart rate variability (Chang et al., 2013). REB is likely to increase the heart rate via peripheral sympathetic properties. While we corrected for physiological noise by regression of the global mean signal, instead of recording heart rate and respiration separately (Glover et al., 2000; Chang and Glover, 2009), such effects of heart rate variability may well be addressed in future studies. Our sample was too small to assess any potential order effects in our crossover design. While in a patients’ population these would have been crucial, we only investigated the drug effects in healthy subjects, where order effects based on any symptom improvement were not expected. In principle, however, these effects are of interest, and our findings may power such an analysis in the future by incorporating the effect sizes observed here.

## Conclusion

Within a double-blind, placebo-controlled, crossover design, we investigated the effects of the noradrenergic antidepressant REB and the dopamine antagonist AMS on resting state activity within a sample of healthy male subjects to examine drug effects independent from disease-related neural alterations. Local resting state behavior was examined by fALFF, whereas interregional characteristics of rs-fMRI were assessed by rs-FC. As measured by fALFF, local rs-activity within regions of the NE-pathway, e.g. the locus coeruleus, was altered by REB and linearly correlated with REB plasma levels. Moreover, REB led to increased rs-FC between regions within the NE-pathway, e.g. the locus coeruleus, tectum, thalamus, amygdala, and hippocampus. AMS increased local rs-activity of regions within the dopaminergic pathway, like the putamen, which is also linearly correlated with AMS plasma levels. Increased rs-FC was also observed between regions of the dopaminergic pathway, comprising the substantia nigra, putamen, thalamus, and amygdala. Thus, our results provide evidence for drug effects on both interregional/global and local neural rs-activity under reboxetine and amisulpride that should be considered in interpreting rs-fMRI data conducted in psychiatric patients.

## Statement of Interest

None.

## Supplementary Material

Table S1
