# Supplementary figures and images for: Local and Global Resting State Activity in the Noradrenergic and Dopaminergic Pathway Modulated by Reboxetine and Amisulpride in Healthy Subjects
Source: Int J Neuropsychopharmacol. 2015 Jul 25;19(2):pyv080. doi: 10.1093/ijnp/pyv080 (PMC4772816; doi:10.1093/ijnp/pyv080)

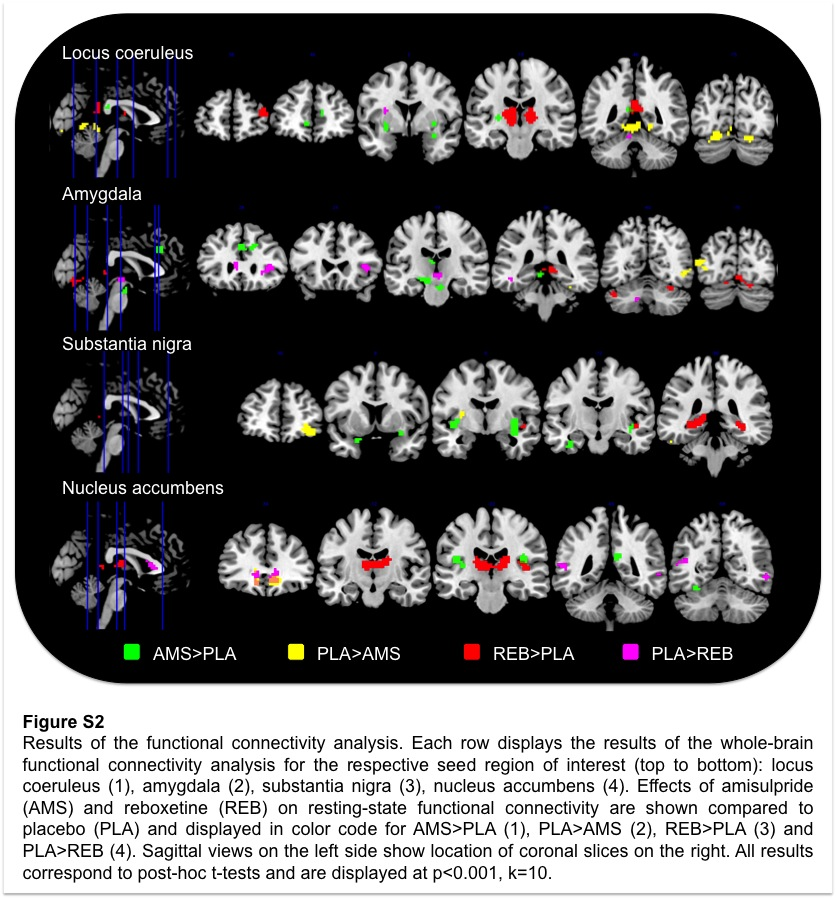

Supplement: Table S1 [file figure_S2_rev.tif]

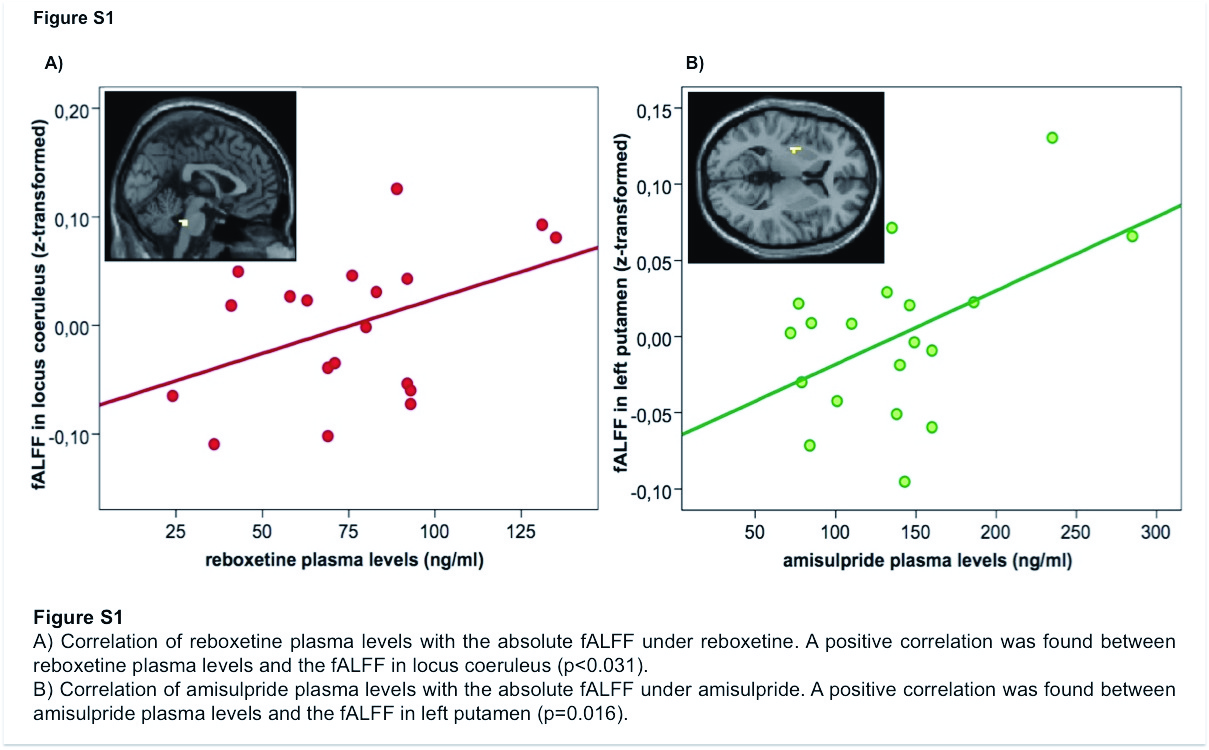

Supplement: Table S1 [file figure_S1_CMYK.tif]
