# Supplementary material for: Local and Global Resting State Activity in the Noradrenergic and Dopaminergic Pathway Modulated by Reboxetine and Amisulpride in Healthy Subjects
Source: Int J Neuropsychopharmacol. 2015 Jul 25;19(2):pyv080. doi: 10.1093/ijnp/pyv080 (PMC4772816; doi:10.1093/ijnp/pyv080)
Supplement: Table S1 [file supplementary_tables.pdf]

Supplementary Tables

Table S1:

	k	z	p-unc.	x,y,z
**Locus coeruleus/tectum**	460	4,952	< 0,000001	[6;-30;-21]
	*	4,566	< 0,00001	[-6;-39;-24]
**cerebellum**	*	4,201	< 0,0001	[9;-51;-15]
**putamen left**	160	4,565	< 0,00001	[-30;-6;-9]
	*	4,024	< 0,0001	[-27;-9;6]
	*	3,690	< 0,001	[-21;-12;18]
**pulvinar right**	17	3,772	< 0,0001	[24;-24;18]
**hypothalamus**	17	3,699	< 0,001	[3;-3;-9]
**sgACC**	10	3,587	< 0,001	[0;30;0]
**anterior insula right**	15	3,377	< 0,001	[48;9;-15]
	*	3,355	< 0,001	[39;18;-21]

Clusters showing a main effect of drug on the frequency amplitude of low frequency fluctuations (fALFF). All clusters are reported at p<0.001, k=10. For the interest of the reader, exact p-values are reported. Regions marked with (*) are part of the previous cluster.

Table S2:

target region	ANOVA-f-test			AMS > PLA			REB > PLA			REB < PLA		
**SUBCORTICAL REGIONS**	z	NV	peak	z	NV	peak	z	NV	peak	z	NV	peak
amygdala right	5,005	a	[33;3;-15]	4,0987	a	[33;3;-18]						
hippocampus left				3,505	23	[-30;-27;-18]						
hippocampus right				3,764	a	[21;-9;-12]	4,042	11	[27;-9;-15]			
medial and dorsal thalamus left	5,667	106	[-12;-18;12]				6,001	265	[-12;-18;12]			
medial and dorsal thalamus right	4,466	57	[15;-18;6]				4,912	a	[15;-18;6]			
	3,194	a	[6;-9;12]									
putamen left				3,924	45	[-30;0;-6]						
				3,545	a	[-27;18;-9]						
				3,548	11	[-24;-21;6]						
putamen right	4,594	62	[33;-3;0]	4,753	75	[33;-3;0]						
**CORTICAL REGIONS**												
dorsal anterior cingulate cortex right				3,373	13	[9;48;9]						
medial occipital cortex left										3,285	18	[-12;-66;0]
lateral occipital cortex left				3,654	15	[-36;-54;18]						
lateral occipital cortex right	3,63	10	[48;-57;3]				3,249	10	[60;-42;-18]	4,182	40	[48;-57;3]
lateral orbitofrontal cortex left							3,786	11	[-27;69;9]			
lateral orbitofrontal cortex right	3,605	18	[39;57;15]				4,075	39	[39;57;15]			
lateral temporal cortex right	4,235	24	[48;12;-24]							4,013	26	[54;9;-18]
	3,74	a	[54;9;-18]									
medial insula right										4,054	11	[-30;6;15]
medial orbitofrontal cortex left							3,668	14	[-9;69;18]			
posterior cingulate cortex left	3,634	16	[-3;-45;15]	3,659	30	[-6;-45;15]	4,038	44	[-3;-45;15]			
				3,4944	a	[-3;-33;21]						
posterior insula left							4,144	20	[-33;-30;9]			
precuneus right										3,613	13	[21;-54;3]
pregenual anterior cingulate cortex left				3,432	11	[-9;45;0]						
				3,251	a	[-6;48;-9]						
**CEREBELLUM**												
cerebellum middle	3,851	16	[6;-45;0]									
cerebellum left	4,186	44	[-21;-78;-15]							3,455	11	[-12;-51;-21]
	3,359	a	[-6;-90;-12]									
cerebellum right	4,799	32	[21;-78;-21]									

Locus coeruleus-seeded connectivity. Main effects of drug (ANOVA-f-test) as well as results of the post-hoc tests for the contrasts AMS>PLA, REB> PLA and REB< PLA are shown in detail (all p<0.001, k=10). Regions marked with ‘a’ are part of the previous cluster. Significant clusters for the contrast AMS<PLA were solely found in the cerebellum.

Table S3:

target region	ANOVA-f-test			AMS > PLA			AMS < PLA			REB > PLA			REB < PLA		
**SUBCORTICAL REGIONS**	z	NV	peak	z	NV	peak	z	NV	peak	z	NV	peak	z	NV	peak
medial thalamus left				3.590	11	[-6;-18;12]									
ventral thalamus	3.895	30	[3;-18;-6]												
midbrain	3.295	a	[9;-9;-6]										3.895	30	[3;-18;-6]
													3.295	a	[9;-9;-6]
hippocampus left	3.889	17	[-27;-6;-33]	4.083	91	[-15;-15;-9]							3.889	17	[-27;-6;-33]
substantia nigra left				4.252	a	[-6;-12;-15]									
substantia nigra right				3.788	a	[6;-15;-21]									
**CORTICAL REGIONS**															
pregenual anterior cingulate cortex left	4.279	11	[-6;30;9]										4.279	11	[-6;30;9]
dorsal anterior cingulate cortex				3.681	43	[0;30;33]									
				3.571	a	[18;27;33]									
anterior insula cortex right	3.976	62	[36;27;6]										3.976	62	[36;27;6]
													3.605	a	[27;33;-3]
													3.410	a	[45;9;3]
medial insula cortex left	3.819	11	[-33;3;-9]										3.819	11	[-33;3;-9]
lateral temporal cortex left	4.202	25	[-45;-33;-9]										4.202	25	[-45;-33;-9]
													3.420	a	[-57;-27;-9]
parahippocampal gyrus left				3.621	11	[-6;-36;-3]				3.820	29	[-6;-39;3]			
parahippocampal gyrus right							3.592	10	[30;-30;-15]	3.827	a	[9;-36;0]			
medial occipital cortex										4.317	60	[3;-78;-9]			
lateral occipital cortex left							3.973	61	[-48;-81;6]						
lateral occipital cortex right							3.458	22	[48;-60;-3]						
**CEREBELLUM**															
cerebellum left	4.129	30		[-15;-54;-42]						4.154	47	[-15;-84;-21]	4.129	30	[-15;-54;-42]
	3.736	a		[-27;-48;-42]						3.636	a	[-12;-72;-15]	3.736	a	[-27;-48;-42]
										3.920	17	[-42;-57;-30]			
cerebellum right	3.976	62	[36;27;6]							3.768	24	[36;-66;-21]			
	3.605	a	[27;33;-3]							3.614	a	[30;-60;-21]			
	3.410	a	[45;9;3]												

Amygdala-seeded connectivity. Main effects of drug (ANOVA-f-test) as well as results of the post-hoc tests for the contrasts AMS>PLA, AMS<PLA, REB> PLA and REB< PLA are shown in detail (all $p<0.001$, k=10). Regions marked with ‘a’ are part of the previous cluster.

Table S4:

target region	ANOVA-f-test			AMS > PLA			REB > PLA			REB < PLA		
**SUBCORTICAL REGIONS**	z	NV	peak	z	NV	peak	z	NV	peak	z	NV	peak
putamen left	5,596	86	[-30;-6;9]	3,795	38	[-36;-6;0]				4,588	15	[-30;-6;9]
	4,353	a	[-36;-6;-3]									
putamen right	4,572	80	[33;0;-6]	4,468	136	[33;0;-6]						
				3,590	a	[27;0;12]						
				3,520	a	[30;0;-18]						
hippocampus left	3,891	10	[-36;-12;-27]	4,131	32	[-33;-12;-27]						
hippocampus right				4,667	13	[33;-18;-18]						
				3,524	a	[-33;-24;-18]						
amygdala left				3,454	20	[-24;9;-21]						
**CORTICAL**												
orbitofrontal cortex right	3,647	10	[33;48;-12]							4,038	47	[33;48;-9]
anterior insula cortex right	3,504	11	[42;15;-21]									
posterior insula cortex right							3,649	18	[45;-15;-6]			
parahippocampal gyrus left	4,644	90	[-21;-39;0]				5,105	113	[-21;-39;0]			
	4,060	a	[-6;-42;3]				3,798	a	[-21;-51;24]			
	3,892	a	[6;-45;0]				3,371	a	[-3;-42;3]			
parahippocampal gyrus right							3,988	28	[33;-39;-3]			
inferior temporal cortex left										3,770	17	[-54;-33;-24]
**CEREBELLUM**												
cerebellum right	4,137	31	[9;-84;-18]									

Substantia nigra-seeded connectivity. Main effects of drug (ANOVA-f-test) as well as results of the post-hoc tests for the contrasts AMS>PLA, REB> PLA and REB< PLA are shown in detail (all $p<0.001$, $k=10$). Regions marked with 'a' are part of the previous cluster. No significant clusters were found for the contrast AMS<PLA.

Table S5:

target region	ANOVA-f-test			AMS > PLA			AMS < PLA			REB > PLA			REB < PLA		
**SUBCORTICAL REGIONS**	z	NV	peak	z	NV	peak	z	NV	peak	z	NV	peak	z	NV	peak
thalamic pulvinar right	5,651	179	[18;-18;18]							5,540	244	[18;-18;18]			
										4,852	a	[9;-15;12]			
thalamic pulvinar left	4,714	a	[-6;-18;12]							5,180	a	[-6;-18;12]			
**CORTICAL**															
pregenual anterior cingulate cortex left	4,910	68	[-12;42;-6]				4,311	78	[-12;42;-6]				5,101	81	[-12;42;-6]
													4,021	a	[-12;33;0]
subgenual anterior cingulate cortex left	4,072	a	[-9;21;-6]				4,553	a	[-9;21;-6]				3,964	a	[-12;30;-12]
	3,611	a	[-12;33;0]				4,065	a	[-12;33;-9]						
subgenual anterior cingulate cortex right	4,400	44	[9;30;-9]				4,557	54	[9;30;-9]				4,024	49	[9;30;-9]
pregenual anterior cingulate cortex right	3,405	a	[15;39;0]										3,769	a	[15;39;0]
posterior cingulate cortex right				4,309	23	[9;-42;21]				3,563	22	[6;-39;9]			
				3,873	14	[6;-27;30]				3,418	a	[-6;-39;6]			
										3,152	a	[-15;-36;3]			
posterior insula right	3,488	11	[39;-21;18]	3,950	37	[39;-21;18]				3,653	24	[39;-24;6]			
posterior insula left				3,651	59	[-39;-27;21]									
				3,621	a	[-33;-27;6]									
				3,474	a	[-30;-27;24]									
fusiform gyrus left				3,793	10	[-30;-57;-18]									
secondary visual cortex left	3,974	22	[-21;-93;6]	4,061	35	[-18;-96;3]				3,932	21	[-21;-93;6]			
				3,845	a	[-27;-87;15]									
lateral occipital cortex left							3,870	14	[-51;-81;15]				4,370	22	[-51;-81;12]
superior temporal gyrus left													3,806	48	[-45;-57;15]
													3,630	a	[-57;-48;9]
medial temporal gyrus left													3,494	a	[-54;-39;9]
medial temporal gyrus right													3,699	10	[-57;-27;-12]
													3,637	22	[54;-57;-3]

Nucleus accumbens-seeded connectivity. Main effects of drug (ANOVA-f-test) as well as results of the post-hoc tests for the contrasts AMS>PLA, REB> PLA and REB< PLA are shown in detail (all p<0.001, k=10). Regions marked with 'a' are part of the previous cluster.
